# Combining Sorafenib and Immunosuppression in Liver Transplant Recipients with Hepatocellular Carcinoma

**DOI:** 10.3390/ph14010046

**Published:** 2021-01-09

**Authors:** Koen G. A. M. Hussaarts, Leni van Doorn, Sander Bins, Dave Sprengers, Peter de Bruijn, Roelof W. F. van Leeuwen, Stijn L. W. Koolen, Teun van Gelder, Ron H. J. Mathijssen

**Affiliations:** 1 Department of Medical Oncology, Erasmus MC Cancer Institute, 3015 CN Rotterdam, The Netherlands; l.vandoorn@erasmusmc.nl (L.v.D.); s.bins@erasmusmc.nl (S.B.); p.debruijn@erasmusmc.nl (P.d.B.); r.w.f.vanleeuwen@erasmusmc.nl (R.W.F.v.L.); s.koolen@erasmusmc.nl (S.L.W.K.); a.mathijssen@erasmusmc.nl (R.H.J.M.); 2 Department of Hepatology, Erasmus MC Cancer Institute, 3015 CN Rotterdam, The Netherlands; d.sprengers@erasmusmc.nl; 3 Department of Hospital Pharmacy, Erasmus MC Cancer Institute, 3015 CN Rotterdam, The Netherlands; t.van_Gelder1@lumc.nl

**Keywords:** HCC, liver transplantation, sorafenib

## Abstract

Hepatocellular carcinoma (HCC) recurrence after liver transplantation occurs in approximately 20% of patients. Most of these patients use immunosuppressant drugs. Meanwhile, patients with HCC recurrence are frequently treated with the small molecule kinase inhibitor (SMKI) sorafenib. However, sorafenib and many immunosuppressants are substrates of the same enzymatic pathways (e.g., CYP3A4), which may potentially result in altered SMKI or immunosuppressant plasma levels. Therefore, we investigated changes in drug exposure of both sorafenib and immunosuppressants over time in four patients with systemic immunosuppressant and sorafenib treatment after HCC recurrence. In this study, sorafenib exposure declined over time during combined treatment with immunosuppressants, while two patients also experienced declining tacrolimus plasma levels. Importantly, patients were unable to increase the sorafenib dose higher than 200 mg b.i.d. without experiencing significant toxicity. We recommend to treat patients using both sorafenib and immunosuppressants with a sorafenib starting dose of 200 mg b.i.d.

## 1. Introduction

Hepatocellular carcinoma (HCC) is the sixth most frequently diagnosed cancer type worldwide and the fourth leading cause of cancer death in the world [1]. Liver transplantation [2] is indicated in patients with localized HCC, with a 5-year survival rate of approximately 70% [3]. Still, HCC recurrence in the transplanted liver occurs in about 20% of patients [3].

After HCC recurrence, one of the most applied therapies is sorafenib, an orally active multi-kinase inhibitor approved for the treatment of HCC, resulting in a median overall survival benefit of 7.4 months [4,5,6,7]. Usually sorafenib is started at a 200 mg b.i.d. dose in this patient group due to expected sorafenib side-effects in patients after liver transplantation and is gradually increased based on toxicity. Patients with HCC recurrence after liver transplantation seem to be more susceptible to sorafenib related side effects. Sorafenib side effects include—among others—gastro-intestinal related side effects (e.g., diarrhea) and cutaneous side effects (e.g., hand-foot skin reaction). These side effects lead to dose reduction or even cessation of sorafenib therapy in 15–77% of the treated patients after liver transplantation [8]. The higher incidence of side effects in patients with a liver transplantation may be due to a pharmacokinetic and/or pharmacodynamic drug–drug interaction with immunosuppressants [9,10,11]. Sorafenib and immunosuppressants have overlapping metabolic pathways, which increases the risk of a drug–drug interaction. Sorafenib is metabolized by CYP3A4 and by UGT1A9, while CYP3A4 is also the most important enzyme in the metabolism of several immunosuppressive drugs (e.g., tacrolimus, MTOR inhibitors) [11,12].

Here, we present a case series of four patients with HCC recurrence after liver transplant using tacrolimus concomitantly with sorafenib which allowed to study a possible drug–drug interaction.

## 2. Results

### 2.1. Case 1

**Figure 1:**
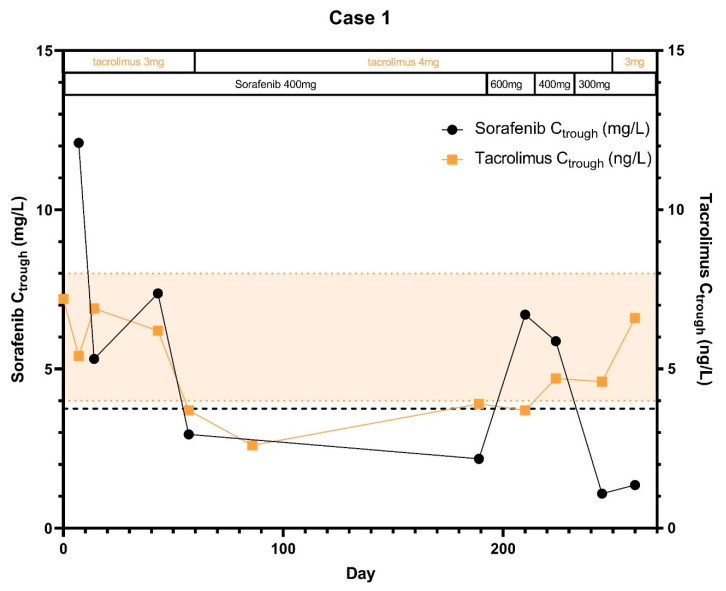
Sorafenib and tacrolimus C_trough_ concentrations over time for Subject 1: The C_trough_ levels are displayed over time after the start of sorafenib treatment. Furthermore the optimal C_trough_ levels of both sorafenib and tacrolimus are provided.

A 62-year old male patient was referred to the department of Medical Oncology for systemic treatment with sorafenib. He had been diagnosed with chronic hepatitis C virus-induced liver cirrhosis before and underwent a liver transplantation for HCC in 2015, followed by tacrolimus monotherapy without previous systemic or local therapy. He had one lesion <5 cm with adequate liver function and no vascular invasion (Barcelona Clinic Liver Cancer (BCLC) score A and Model for End-Stage Liver Disease (MELD)-score was 18). His hepatitis C was treated with ledipasvir, daclatasvir and ribavirin. At start of the study and during hospital admissions patient used loperamide, losartan, metformin and metoprolol as concomitant medication. In June 2017, sorafenib 200 mg b.i.d. was started after HCC recurrence with pulmonary metastases, at which time tacrolimus was dosed at 3 mg once daily providing a tacrolimus trough concentration (C_trough_) of 5.9 μg/L (reference: 4–8 μg/L). The AUC_0–7.5h_ of sorafenib was 2.1% higher at day 14 compared to day 7, while the sorafenib C_max_ was 24% lower (Table 1). In general, both sorafenib and tacrolimus trough levels showed a relevant decrease in the first months of treatment, up to a 90% decrease for sorafenib plasma trough levels compared to the baseline trough level and up to 64% for tacrolimus (Figure 1).

The tacrolimus dose was increased to 4 mg once daily (q.d.) in August 2017, in an attempt to maintain adequate tacrolimus concentrations. As a result, tacrolimus levels increased, while sorafenib levels further decreased. Therefore, also the sorafenib dose was increased with 50% to 200 mg in the morning and 400 mg in the evening in December 2017, after which also the sorafenib C_trough_ increased. Due to CTCAE grade 3 liver toxicity, the sorafenib dose had to be reduced again to 200 mg b.i.d. at first and to 300 mg q.d. (400 mg one day and 200 mg the other) in February 2018. Subsequently, sorafenib concentrations decreased and tacrolimus concentrations further increased. Sorafenib was stopped in May 2018 after progressive disease was noticed at the CT scan.

### 2.2. Case 2

**Figure 2:**
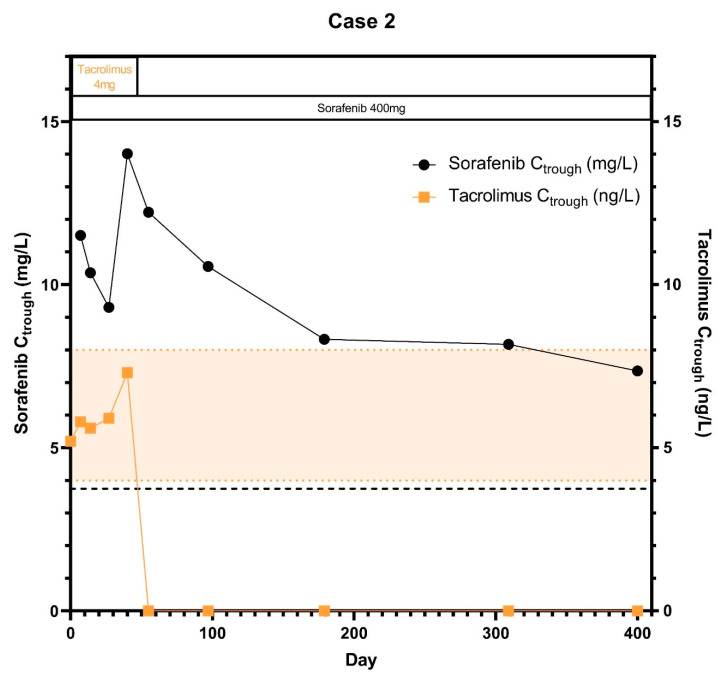
Sorafenib and tacrolimus C_trough_ concentrations over time for Subject 2: The C_trough_ levels over time after the start of sorafenib treatment. Furthermore the optimal C_trough_ levels of both sorafenib and tacrolimus are provided.

A 70-year old female with alcohol induced liver cirrhosis was diagnosed with HCC in 2009, which was at first successfully treated with trans-arterial chemo-embolization (TACE). She had one lesion <5 cm with adequate liver function and no vascular invasion (MELD score: 6), for which she underwent a liver transplantation in January 2011. She developed disease recurrence with pulmonary metastases in 2018, after which she was referred to the department of Medical Oncology for systemic treatment with sorafenib, which was started at a 200 mg b.i.d. dose in July 2018. Patient had no signs of liver fibrosis and had a normal liver function when sorafenib was started. Next to tacrolimus and sorafenib patient used hydrochlorothiazide, losartan and oxazepam concomitantly during start of the study and the hospital admission days. Before start of sorafenib, the tacrolimus dose was 4 mg daily and tacrolimus C_trough_ was 5.2 μg/L. On day 14, AUC_0–7.5h_ and C_max_ of sorafenib were respectively 0.9% and 22.1% higher than at day 7 (Table 1). Sorafenib C_trough_ remained stable during the first 2 weeks of concomitant treatment with tacrolimus but generally declined over time (Figure 2). Hereafter, in August 2018, immunosuppressant therapy was stopped completely by the treating gastroenterologist and sorafenib concentrations further decreased over time. In August 2019, this patient had proven progressive disease and sorafenib was stopped after 19 months of treatment in which there was already a slight progression of disease over time.

### 2.3. Case 3

**Figure 3:**
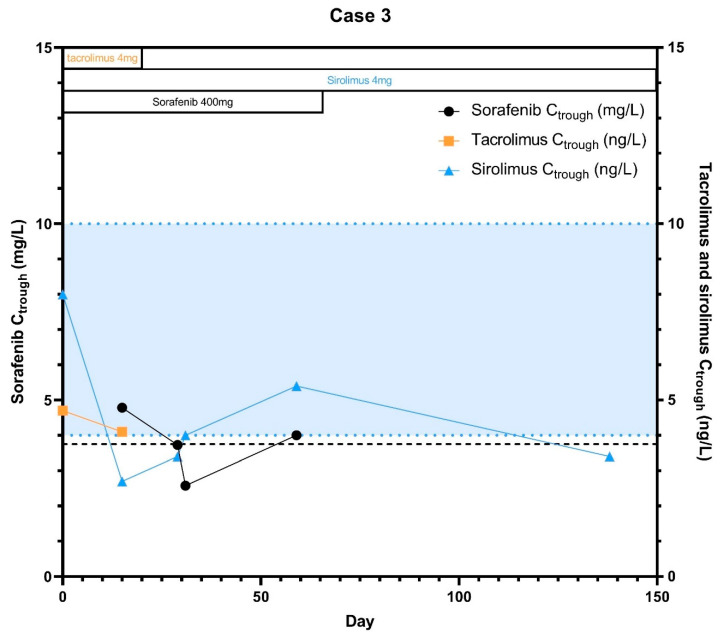
Sorafenib and tacrolimus C_trough_ concentrations over time for Subject 3: The C_trough_ levels are displayed over time after the start of sorafenib treatment. Furthermore the optimal C_trough_ levels of both sorafenib and sirolimus are provided. Case 3 was initially treated with both sirolimus and tacrolimus but stopped tacrolimus short after start of sorafenib as was shown in this figure.

A 65-year old male patient with chronic hepatitis C virus-induced liver cirrhosis was diagnosed with HCC for which he received a liver transplantation in 2018. As transplantation indication he initially had one lesion <5 cm with adequate liver function but with vascular invasion (tumor thrombus), which was first treated with transarterial radioembolization after which there was complete resolvement of the thrombus (BCLC-score C and MELD-score was 6 at time of transplantation). His hepatitis C was treated with peginterferon and ribavirin in 2003, after which there was complete remission. At start of the study and during hospital admissions patient used clopidogrel, temazepam, pravastatin, oxycodon, ursodeoxycholicacid and pantoprazole as concomitant medication. Immunosuppressive treatment consisted of mycophenolate mofetil (MMF) 1000 mg b.i.d. and tacrolimus (4 mg b.i.d., which was later reduced to 4 mg q.d.). Later, the patient switched from MMF to sirolimus (2 mg q.d.) due to livertoxicity. In April 2019, the patient had a recurrence of disease after which sorafenib was started in a dose of 200 mg b.i.d. Both tacrolimus and sirolimus concentrations were adequate at baseline (C_trough_ = 4.7 μg/L and C_trough_ = 8.0 μg/L, respectively). At day eight of sorafenib treatment, tacrolimus was stopped by the gastroenterologist according to physician’s choice and the patient continued with sirolimus monotherapy. After cessation of tacrolimus, the sorafenib concentration initially decreased and remained relatively stable until disease progression, which was also the case for sirolimus concentration (Figure 3). AUC_0–7.5h_ and C_max_ of sorafenib decreased with 40.0% and 37.3% respectively at day 14 compared to day 7 (Table 1). After just 2 months of treatment, this patient had disease progression after which sorafenib treatment was stopped and best supportive care was started. After stopping sorafenib therapy, the sirolimus plasma levels further decreased with 42.6% compared to the latest C_trough_ with the combination therapy.

### 2.4. Case 4

**Figure 4:**
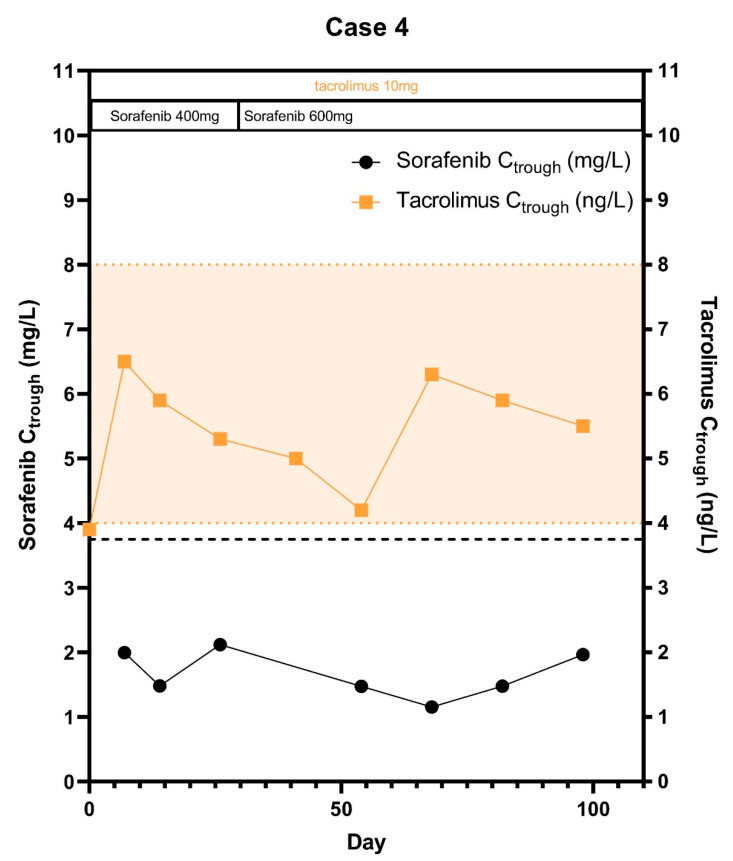
Sorafenib and tacrolimus C_trough_ concentrations over time for Subject 4: The C_trough_ levels are displayed over time after the start of sorafenib treatment. Furthermore the optimal C_trough_ levels of both sorafenib and tacrolimus are provided.

A 69-year old male with alcohol-induced liver cirrhosis was diagnosed with HCC and underwent a liver transplantation in March 2019. He initially did not fit into the Milan criteria, because he had three lesions of which one lesion was more than 3 cm. This lesion was treated with transarterial chemoembolization after which he fell inside the Milan criteria (BCLC-score: A, MELD-score was 11). Due to rapid disease recurrence, this patient started with sorafenib in June 2019. At time of the start of the study and during hospital admissions patient used tiotropium, perindopril, tamsulosin, insulin, oxazepam, pantoprazole, metformin, salbutamol, prednisolone and metoprolol as additional comedication. His dose of tacrolimus was 10 mg q.d., with a baseline tacrolimus C_trough_ of 3.9 μg/L. Sorafenib exposure was remarkably lower at day 14 than at day 7, as the AUC_last_ decreased with 44% and C_max_ with 62% respectively (Table 1). During the further treatment, sorafenib showed a decrease in plasma trough levels over time despite a dose increase to 200 mg once daily and 400 mg once daily (Figure 4). On the other hand, the tacrolimus plasma concentration remained relatively stable over time. In October 2019 sorafenib was stopped due to progression of disease.

## 3. Discussion

In this study, we present the first case series of patients treated with sorafenib for HCC recurrence after liver transplantation investigating both sorafenib and immunosuppressant plasma concentration over time. In all four patients the plasma pharmacokinetics of both immunosuppressants and sorafenib were longitudinally monitored until sorafenib discontinuation. Sorafenib plasma concentrations (C_trough_) decreased over time in every case, even after discontinuation of tacrolimus in two of four cases. Long-term decrease in TKI exposure is a recognized phenomenon and we cannot distill a consequent pharmacokinetic influence of immunosuppression on the gradually decreased sorafenib exposure from our results. This decline in sorafenib exposure may be induced by autoinduction of CYP3A4, which results in declining plasma levels over time as was demonstrated for imatinib before [13,14]. However, variation in immunosuppression concentrations was not structural (two patients showed a decline in immunosuppression plasma exposure, while the other two patients showed opposite effects), which makes structural CYP3A4 induction less likely [15]. Potentially sorafenib non-adherence may have contributed to the decline in sorafenib concentrations over time, since patient adherence was only questioned when meeting the treating oncologist. Moreover, about 50% of patients on long-term oral anticancer drug therapy tend to be non-adherent to their treatment resulting in a diminished therapy efficacy and (unexplained) decline in plasma levels [16].

Although a clear pharmacokinetic interaction of tacrolimus and sorafenib was not found, a sorafenib dose increment to 600 mg daily led to severe hepatotoxicity in case 1. Although sorafenib concentrations increased prior to occurrence of the adverse events, the absolute concentrations of sorafenib did not exceed those measured at start of therapy, which contradicts a sole pharmacokinetic explanation. Both laboratory and imaging findings did not show other causes of hepatotoxicity (e.g., viral hepatitis) and other side -effects in our patients. Therefore, it is likely that an additional pharmacodynamic mechanism is causing the high incidence of sorafenib-induced toxicity after liver transplantation. In this study there were no acute rejections, but patients experienced many side-effects with increasing sorafenib dose. As mentioned before, sorafenib toxicity rates are higher in patients treated with immunosuppression. In several studies, a high incidence of sorafenib dose reduction or discontinuation (15–77%) has been reported in patients with HCC after liver transplantation when starting with a 400 mg b.i.d. dose [17,18,19]. However, the proportion of patients in need of dose reduction or discontinuation seemed to be lower in Asian population studies, suggesting a possible genetic difference [4]. Based on these observations, starting with a lower than regular sorafenib dose seems to be justified in most patients, since the majority of patients required a dose reduction and most patients did not experience significant toxicity at lower dosing levels [19]. Although it is currently no standard of care, this strategy may also improve patient adherence in patients without a previous liver transplantation, as a result of lower toxicity rates compared to the 400 mg starting dose. Unfortunately none of these studies investigated sorafenib or immunosuppressant pharmacokinetics. Because sorafenib plasma trough concentrations showed a decrease in our patients, the underlying mechanism of this increase in side effects most likely is of pharmacodynamic origin. Moreover, the immunocompromised status of these patients may be related to an increased incidence of side effects in post liver transplantation patients. However, the exact mechanism remains unknown.

Moreover, an important aspect in the immunosuppressant treatment of patients with HCC recurrence after liver transplantation is the class of immunosuppressants used. Latest evidence suggest survival benefit of treatment with mammalian target of rapamycin (mTOR) inhibitors compared to calcineurin inhibitors like tacrolimus especially when used with sorafenib [6]. However, general consensus on this topic is not yet reached and alternative therapies, such as lowering immunosuppressant dosing as much as possible, are used in clinical practice. All the patients in this study are treated according to the national treatment guidelines in the Netherlands. From a pharmacokinetic point of view most CNIs have similar pharmacokinetic properties compared to mTOR inhibitors the effects seen in this case-series may also be applied for these class of immunosuppressants. Moreover, additional treatment strategies for hepatocellular carcinoma patients are emerging, among which immunotherapy regimens. However, this is no option in patient with a liver transplantation, because of the major risk of transplant rejection [20]. Therefore TKI treatment remains the standard treatment in these patients despite this new developments. Next to sorafenib, alternative dosing strategies for other TKIs such as cabozantinib, regorafenib, and imatinib, were suggested before. However evidence in liver transplantation patients is lacking [21,22,23]. In transplanted patients with a malignancy in general, physicians attempt to lower the overall immunosuppressive load as much as possible, but it is very difficult to define the lower threshold of the target range for individual patients. Sometimes with trial and error dosages are reduced stepwise, while liver function is monitored closely. In the second case of our series the immunosuppression was stopped completely, and patient and medical team were fortunate that this did not result in a rejection episode.

Several lessons can be learned from this case series. First of all, there is currently a lack of knowledge in the management of the combination of sorafenib and tacrolimus. Oncologists often determine the sorafenib starting dose on the basis of personal experience with this treatment combination. Overall, there is a decrease in sorafenib plasma levels over time, even when it is not combined with tacrolimus. Due to an increased risk of side effects in patients with a liver transplantation [9], and based on the high incidence of side effects with higher sorafenib doses we would recommend to start treatment with a reduced daily dose of 200 mg b.i.d. [4]. Based on tolerability, the dose can then gradually be escalated. Moreover, a daily sorafenib dose of 200 mg b.i.d. has demonstrated to be an effective dosing strategy, which indicates a possible overdosing in most patients treated with sorafenib [24].

## 4. Materials and Methods

In all four patients serial blood samples for the determination of both sorafenib and tacrolimus have been taken as part of usual clinical care, for patient safety reasons. None of these patients used additional interacting comedication. Blood samples were taken at day 7 and 14 after the start of sorafenib for the determination of sorafenib area under the curve (AUC_0–7.5_) and C_max_, at time point t = 0 h (before intake of sorafenib) as well as 2, 4, and 7.5 h after intake of sorafenib. At timepoint t = 0 h, blood was also taken for the determination of tacrolimus C_trough._ Next, both tacrolimus and sorafenib C_trough_ were determined on a regular basis at the outpatient clinic. All patients gave written consent for the use of these samples and clinical data for scientific purposes, including this publication.

## 5. Conclusions

In conclusion, the interaction between sorafenib and immunosuppressive drugs is clinically relevant in view of the high toxicity rates compared to patients without a liver transplantation. More research is needed to investigate the pharmacokinetic aspects of this drug–drug interaction.

## Figures and Tables

**Table 1:** AUC_0–7.5h_ and C_max_ of each individual case.

	Day 7		Day 14			
Case	AUC_0–7.5h_ Sorafenib (mg*h/L)	C_max_ Sorafenib (mg/L)	AUC_0–7.5h_ Sorafenib (mg*h/L)	C_max_ Sorafenib (mg/L)	RD AUC_0–7.5h_ (%)	RD (%) C_max_
1	33.4	8.5	34.1	6.4	+2.1	−24.4
2	47.8	8.7	48.2	10.7	+0.9	+22.1
3	37.6	6.3	22.6	4.0	−37.3	−40.0
4	24.9	6.0	13.9	2.3	−62.1	−44.0

All patients used sorafenib 200 mg b.i.d. Abbreviations: AUC = area under the plasma curve, RD = relative difference; C_max_ = maximum concentration; RD = relative difference.

## Data Availability

The data presented in this study are available on request from the corresponding author. The data are not publicly available due to privacy considerations.

## References

[1] Bray F., Ferlay J., Soerjomataram I., Siegel R.L., Torre L.A., Jemal A. (2018). Global cancer statistics 2018: GLOBOCAN estimates of incidence and mortality worldwide for 36 cancers in 185 countries. CA A Cancer J. Clin..

[2] Iavarone M., Invernizzi F., Czauderna C., Sanduzzi-Zamparelli M., Bhoori S., Amaddeo G., Manini M.A., López M.F., Anders M., Pinter M. (2019). Preliminary experience on safety of regorafenib after sorafenib failure in recurrent hepatocellular carcinoma after liver transplantation. Am. J. Transplant..

[3] Zimmerman M.A., Ghobrial R.M., Tong M.J., Hiatt J.R., Cameron A.M., Hong J., Busuttil R.W. (2008). Recurrence of Hepatocellular Carcinoma Following Liver Transplantation: A Review of Preoperative and Postoperative Prognostic Indicators. Arch. Surg..

[4] Kang S.H., Cho H., Cho E.J., Lee J.H., Yu S.J., Kim Y.J., Yi N.J., Lee K.W., Suh K.S., Yoon J.H. (2018). Efficacy of Sorafenib for the Treatment of Post-Transplant Hepatocellular Carcinoma Recurrence. J. Korean Med. Sci..

[5] Sposito C., Mariani L., Germini A., Reyes M.F., Bongini M., Grossi G., Bhoori S., Mazzaferro V. (2013). Comparative efficacy of sorafenib versus best supportive care in recurrent hepatocellular carcinoma after liver transplantation: A case-control study. J. Hepatol..

[6] Verna E.C., Patel Y.A., Aggarwal A., Desai A.P., Frenette C., Pillai A.A., Salgia R., Seetharam A., Sharma P., Sherman C. (2020). Liver transplantation for hepatocellular carcinoma: Management after the transplant. Am. J. Transplant..

[7] Berenguer M., Burra P., Ghobrial M., Hibi T., Metselaar H., Sapisochin G., Bhoori S., Man N.K., Mas V., Ohira M. (2020). Posttransplant management of recipients undergoing liver transplantation for hepatocellular carcinoma. Working Group Report from the ILTS Transplant Oncology Consensus Conference. Transplantation.

[8] Li Y., Gao Z.H., Qu X.J. (2015). The adverse effects of sorafenib in patients with advanced cancers. Basic Clin. Pharmacol. Toxicol..

[9] Staufer K., Fischer L., Seegers B., Vettorazzi E., Nashan B., Sterneck M. (2012). High toxicity of sorafenib for recurrent hepatocellular carcinoma after liver transplantation. Transpl. Int..

[10] Kim R., El-Gazzaz G., Tan A., Elson P., Byrne M., Chang Y.D., Aucejo F. (2010). Safety and feasibility of using sorafenib in recurrent hepatocellular carcinoma after orthotopic liver transplantation. Oncology.

[11] Takahara T., Nitta H., Hasegawa Y., Itou N., Takahashi M., Wakabayashi G. (2011). Using sorafenib for recurrent hepatocellular carcinoma after liver transplantation—Interactions between calcineurin inhibitor: Two case reports. Transplant. Proc..

[12] Bins S., van Doorn L., Phelps M.A., Gibson A.A., Hu S., Li L., Vasilyeva A., Du G., Hamberg P., Eskens F.A.L.M. (2017). Influence of OATP1B1 Function on the Disposition of Sorafenib-beta-D-Glucuronide. Clin. Transl. Sci..

[13] Filppula A.M., Laitila J., Neuvonen P.J., Backman J.T. (2012). Potent mechanism-based inhibition of CYP3A4 by imatinib explains its liability to interact with CYP3A4 substrates. Br. J. Pharmacol..

[14] Eechoute K., Fransson M.N., Reyners A.K., De Jong F.A., Sparreboom A., Van Der Graaf W.T.A., Friberg L.E., Schiavon G., Wiemer E.A.C., Verweij J. (2012). A long-term prospective population pharmacokinetic study on imatinib plasma concentrations in GIST patients. Clin. Cancer Res..

[15] Hussaarts K., Veerman G.D.M., Jansman F.G.A., van Gelder T., Mathijssen R.H.J., van Leeuwen R.W. (2019). Clinically relevant drug interactions with multikinase inhibitors: A review. Ther. Adv. Med. Oncol..

[16] Timmers L., Boons C.C.L.M., Verbrugghe M., Bemt B.J.F.V.D., Van Hecke A., Hugtenburg J.G. (2017). Supporting adherence to oral anticancer agents: Clinical practice and clues to improve care provided by physicians, nurse practitioners, nurses and pharmacists. BMC Cancer.

[17] De Simone P., Crocetti L., Pezzati D., Bargellini I., Ghinolfi D., Carrai P., Leonardi G., Della Pina C., Cioni D., Pollina L. (2014). Efficacy and Safety of Combination Therapy With Everolimus and Sorafenib for Recurrence of Hepatocellular Carcinoma After Liver Transplantation. Transplant. Proc..

[18] Weinmann A., Niederle I.M., Koch S., Hoppe-Lotichius M., Heise M., Düber C., Schuchmann M., Otto G., Galle P., Wörns M.A. (2012). Sorafenib for recurrence of hepatocellular carcinoma after liver transplantation. Dig. Liver Dis..

[19] Castelli G., Burra P., Giacomin A., Vitale A., Senzolo M., Cillo U., Farinati F. (2014). Sorafenib use in the transplant setting. Liver Transpl..

[20] Pinter M., Scheiner B., Peck-Radosavljevic M. (2020). Immunotherapy for advanced hepatocellular carcinoma: A focus on special subgroups. Gut.

[21] Ramadori G., Füzesi L., Grabbe E., Pieler T., Armbrust T. (2004). Successful treatment of hepatocellular carcinoma with the tyrosine kinase inhibitor imatinib in a patient with liver cirrhosis. Anticancer Drugs..

[22] Lin A.Y., Fisher G.A., So S., Tang C., Levitt L. (2008). Phase II study of imatinib in unresectable hepatocellular carcinoma. Am. J. Clin. Oncol..

[23] Delos Santos S., Udayakumar S., Nguyen A., Ko Y.J., Berry S., Doherty M., Chan K.K. (2020). A systematic review and network meta-analysis of second-line therapy in hepatocellular carcinoma. Curr. Oncol..

[24] Siegel A.B., El-Khoueiry A.B., Finn R.S., Guthrie K.A., Goyal A., Venook A.P., Blanke C.D., Verna E.C., Dove L., Emond J.C. (2015). Phase I trial of sorafenib following liver transplantation in patients with high-risk hepatocellular carcinoma. Liver Cancer.

